# Supplementary material for: CD8+ T cell stemness precedes post-intervention control of HIV viraemia
Source: Nature. 2025 Dec 1;650(8100):196–204. doi: 10.1038/s41586-025-09932-w (PMC12872466; doi:10.1038/s41586-025-09932-w)
Supplement: Supplementary file 1 — Reporting Summary [file 41586_2025_9932_MOESM1_ESM.pdf]

nature portfolio

Corresponding author(s): David R. Collins

Last updated by author(s): 10/10/2025

Reporting Summary

Nature Portfolio wishes to improve the reproducibility of the work that we publish. This form provides structure for consistency and transparency in reporting. For further information on Nature Portfolio policies, see our Editorial Policies and the Editorial Policy Checklist.

Statistics

For all statistical analyses, confirm that the following items are present in the figure legend, table legend, main text, or Methods section.

n/a	Confirmed	
☐	☒	The exact sample size (*n*) for each experimental group/condition, given as a discrete number and unit of measurement
☐	☒	A statement on whether measurements were taken from distinct samples or whether the same sample was measured repeatedly
☐	☒	The statistical test(s) used AND whether they are one- or two-sided<br>*Only common tests should be described solely by name; describe more complex techniques in the Methods section.*
☒	☐	A description of all covariates tested
☐	☒	A description of any assumptions or corrections, such as tests of normality and adjustment for multiple comparisons
☐	☒	A full description of the statistical parameters including central tendency (e.g. means) or other basic estimates (e.g. regression coefficient) AND variation (e.g. standard deviation) or associated estimates of uncertainty (e.g. confidence intervals)
☐	☒	For null hypothesis testing, the test statistic (e.g. *F*, *t*, *r*) with confidence intervals, effect sizes, degrees of freedom and *P* value noted<br>*Give P values as exact values whenever suitable.*
☒	☐	For Bayesian analysis, information on the choice of priors and Markov chain Monte Carlo settings
☐	☒	For hierarchical and complex designs, identification of the appropriate level for tests and full reporting of outcomes
☐	☒	Estimates of effect sizes (e.g. Cohen's *d*, Pearson's *r*), indicating how they were calculated

Our web collection on statistics for biologists contains articles on many of the points above.

Software and code

Policy information about availability of computer code

Data collection	IFNG elispot data collection was performed using CTL ImmunoSpot Analyzer Pro version 7.0.38.16. Flow cytometric data collection and FACS were performed using BD FACSDiva version 9.2.
Data analysis	Flow cytometric data analyses were performed using FlowJo version 10.10.0. Statistical analyses were performed using GraphPad Prism version 10.4 and R version 4.3.1. Single-cell multiomics data analyses were performed using R version 4.3.1, cellranger version 9.0.0, bcl2fastq version 2.20, seurat version 5.3.0, tmod version 0.46.2, and GSNA version 0.1.4.9. Data visualizations were prepared using ggplot2 version 3.5.2 and Adobe Illustrator version 29.8.2.

For manuscripts utilizing custom algorithms or software that are central to the research but not yet described in published literature, software must be made available to editors and reviewers. We strongly encourage code deposition in a community repository (e.g. GitHub). See the Nature Portfolio guidelines for submitting code & software for further information.

Data

Policy information about availability of data

All manuscripts must include a data availability statement. This statement should provide the following information, where applicable:

- Accession codes, unique identifiers, or web links for publicly available datasets
- A description of any restrictions on data availability
- For clinical datasets or third party data, please ensure that the statement adheres to our policy

Full single-cell multiomics data are available via the NCBI Gene Expression Omnibus (GEO) via accession number GSE294440. The GRCh38 reference genome is available at NCBI GenBank via accession number GCA_000001405.15. MSigDB gene set references can be obtained from https://data.broadinstitute.org/gsea-msigdb/msigdb/release/7.5.1/. The remaining data are included within the manuscript and supplemental materials.

Research involving human participants, their data, or biological material

Policy information about studies with human participants or human data. See also policy information about sex, gender (identity/presentation), and sexual orientation and race, ethnicity and racism.

Reporting on sex and gender	Biological sex of each participant is reported in Table 1 as previously published for each parent trial.
Reporting on race, ethnicity, or other socially relevant groupings	Race (American Indian, Black, or White) and ethnicity (Hispanic or not Hispanic) of each participant are reported in Table 1 as previously published for each parent trial.
Population characteristics	Age, class-I HLA genotypes, and clinical histories related to HIV are reported for each participant in Table 1 as previously published for each parent trial.
Recruitment	This study includes only secondary use of previously collected samples.
Ethics oversight	Secondary use protocols were approved by the Mass General Brigham Human Research Committee

Note that full information on the approval of the study protocol must also be provided in the manuscript.

Field-specific reporting

Please select the one below that is the best fit for your research. If you are not sure, read the appropriate sections before making your selection.

☒ Life sciences ☐ Behavioural & social sciences ☐ Ecological, evolutionary & environmental sciences

For a reference copy of the document with all sections, see nature.com/documents/nr-reporting-summary-flat.pdf

Life sciences study design

All studies must disclose on these points even when the disclosure is negative.

Sample size	Sample sizes were constrained by specimen and reagent availability.
Data exclusions	Only participants who received intervention were included in the analyses. Participants/responses for which only one longitudinal sample was measured were excluded from longitudinal statistical comparisons. Doublets were excluded from multimodal single-cell analyses based on hashing and tetramer oligonucleotides. Cells with TCRs that occurred only once and cells for which TCR sequences were not detected were excluded from TCR clonotypic analyses.
Replication	Proliferation assays were confirmed in triplicate and averaged. Metrics were also repeated across longitudinal samples for each participant. The precise number of biological replicates is specified for each experiment in the figure legends and each data point is displayed in the figures. Representative data are only shown adjacent to the corresponding full data set for illustrative purposes. Further replication beyond those listed here were prohibited by limited specimen availability.
Randomization	This manuscript reports secondary analyses of specimens from previous trials. Experimental groups (PIC, PINC) were determined based on the presence or absence of prolonged virologic control without resumption of ART, as previously reported by each parent trial. Longitidinal samples (pre, post) were pre-determined based on which samples were collected prior to or following intervention in the parent trials. Viremia as a potential covariate was controlled by inclusion only of samples without detectable HIV viremia. Demographics are summarized in Table 1. Due to limited participant numbers, covariate modeling or controlling for additional potential covariates was not feasible.
Blinding	As this manuscript reports secondary analyses of specimens from previous trials, formal blinding was not part of the study design.

Reporting for specific materials, systems and methods

We require information from authors about some types of materials, experimental systems and methods used in many studies. Here, indicate whether each material, system or method listed is relevant to your study. If you are not sure if a list item applies to your research, read the appropriate section before selecting a response.

Materials & experimental systems

n/a	Involved in the study
☐	☒ Antibodies
☒	☐ Eukaryotic cell lines
☒	☐ Palaeontology and archaeology
☒	☐ Animals and other organisms
☒	☐ Clinical data
☒	☐ Dual use research of concern
☒	☐ Plants

Methods

n/a	Involved in the study
☒	☐ ChIP-seq
☐	☒ Flow cytometry
☒	☐ MRI-based neuroimaging

Antibodies

Antibodies used: anti-IFN-g, clone DK1, Mabtech, cat# 3420-2A, lot# 161; anti-CD3, clone OKT3, Biolegend, cat# 317326, lot# B407799; anti-CD28, clone CD28.2, Biolegend, cat# 302934, lot# B374639; anti-IFN-g, clone B6-1, Mabtech, cat# 3420-2A, lot#161; AlexaFluor700-anti-CD3, clone SK7, Biolegend, cat# 344822, lot# B420037; APC-anti-CD8, clone RPA-T8, Biolegend, cat# 301014, lot# B386144; BV605-anti-CD3, clone UCHT1, Biolegend, cat# 300460, lot# B430690; BUV395-anti-CD8, clone RPA-T8, BD Biosciences, cat# 563795, lot# 4292914; BV711-anti-CD4, clone RPA-T4, Biolegend, cat# 300558, lot# B420968; RB705-anti-CD3, clone UCHT1, BD Biosciences, cat# 570237, lot# 3229245; BV711-anti-CD8, clone RPA-T8, Biolegend, cat# 301044, lot# B425053; BUV395-anti-CD45RA, clone HI100, BD Biosciences, cat# 740298, lot# 5091519; RB780-anti-CD62L, clone DREG-56, BD Biosciences, cat# 569211, lot# 4200635; PE-Dazzle594-anti-CD38, clone HB-7, Biolegend, cat# 356630, lot# B406413; BUV805-anti-HLA-DR, clone G46-6, BD Biosciences, cat# 568335, lot# 4178322; PE-anti-perforin, clone B-D48, Biolegend, cat# 353304, lot# B397495; FITC-anti-granzyme B, clone GB11, Biolegend, cat# 515403, lot# B397296; BV421-anti-Ki-67, clone Ki-67, Biolegend, cat# 350506, lot# B356738; BV711-anti-CD8, clone RPA-T8, Biolegend, cat# 301044, lot# B425053; Total-Seq C Human Universal Cocktail v2.0, Biolegend, cat# 399910, lot# B408342; Total-Seq C anti-human hashtags 1-18, clone LNH-94/2M2, Biolegend, cat# 394661-394693, lot# B344497

Validation: Species reactivity and suitability for each application were validated by the commercial suppliers (Biolegend, BD Biosciences, Mabtech) for each antibody, with quality control certification provided for each lot.

Plants

Seed stocks: *Report on the source of all seed stocks or other plant material used. If applicable, state the seed stock centre and catalogue number. If plant specimens were collected from the field, describe the collection location, date and sampling procedures.*

Novel plant genotypes: *Describe the methods by which all novel plant genotypes were produced. This includes those generated by transgenic approaches, gene editing, chemical/radiation-based mutagenesis and hybridization. For transgenic lines, describe the transformation method, the number of independent lines analyzed and the generation upon which experiments were performed. For gene-edited lines, describe the editor used, the endogenous sequence targeted for editing, the targeting guide RNA sequence (if applicable) and how the editor was applied.*

Authentication: *Describe any authentication procedures for each seed stock used or novel genotype generated. Describe any experiments used to assess the effect of a mutation and, where applicable, how potential secondary effects (e.g. second site T-DNA insertions, mosiacism, off-target gene editing) were examined.*

Flow Cytometry

Plots

Confirm that:

☒ The axis labels state the marker and fluorochrome used (e.g. CD4-FITC).

☒ The axis scales are clearly visible. Include numbers along axes only for bottom left plot of group (a 'group' is an analysis of identical markers).

☒ All plots are contour plots with outliers or pseudocolor plots.

☒ A numerical value for number of cells or percentage (with statistics) is provided.

Methodology

Sample preparation: Cryopreserved PBMCs were thawed at 37 C and rested overnight in RPMI + 10% FBS prior to each assay.

Instrument: Data were collected using BD FACSSymphony A5, LSR-II, and FACSAria instruments.

Software: Collection was performed using BD FACSDiva. Analysis was performed using FlowJo.

Cell population abundance	Abundances of each cell population/subpopulation are reported for all flow cytometry and multiomics data as frequencies in the figures, extended figures, and supplementary data.
Gating strategy	Intact live CD8+ cells were gated on forward and side scatter, viability dye, and CD8. Elimination assay data were pre-gated on intact, live, CTV+ target cells. Gates are represented in manuscript figures.

☒ Tick this box to confirm that a figure exemplifying the gating strategy is provided in the Supplementary Information.
